# *Acanthamoeba* profilin elicits allergic airway inflammation in mice

**DOI:** 10.1371/journal.pntd.0006979

**Published:** 2018-12-17

**Authors:** So Myung Song, Shin Ae Kang, Hye Kyung Park, Dong Hee Kim, So Young Park, Se Bok Jang, Hak Sun Yu

**Affiliations:** 1 Department of Parasitology School of Medicine, Pusan National University, Yangsan, Gyeongsangnam-do, South Korea; 2 Department of Internal Medicine, School of Medicine, Pusan National University, Yangsan, Gyeongsangnam-do, South Korea; 3 Department of Nursing, College of Nursing, Pusan National University, Yangsan, Gyeongsangnam-do, South Korea; 4 Department of Molecular Biology, College of Natural Sciences, Pusan National University, Busan, South Korea; Institute of Tropical Medicine (NEKKEN), Nagasaki University, JAPAN

## Abstract

**Background:**

In previous studies, we suggested that *Acanthamoeba* is a new aero-allergen and that patients who showed positive results for the skin-prick test response to *Acanthamoeba* cross-reacted with several pollen allergens. Additionally, patients with common antibodies reacted to the 13–15 kDa *Acanthamoeba* unknown allergen.

**Objective:**

We examined whether profilin of *Acanthamoeba* is a human airway allergic agent because of its molecular weight.

**Methods:**

We expressed recombinant Ac-PF (rAc-PF) protein using an *Escherichia coli* expression system and evaluated whether Ac-PF is an airway allergic agent using an allergic airway inflammation animal model.

**Results:**

Airway hyperresponsiveness was increased in rAc-PF-inoculated mice. The number of eosinophils and levels of Th2 cytokines, interleukin (IL)-4, IL-5, and IL-13 were increased in the bronchial alveolar lavage fluid of rAc-PF-treated mice. The lungs of the rAc-PF-treated mice group showed enhanced mucin production and metaplasia of lung epithelial cells and goblet cells.

**Conclusion:**

In this study, we demonstrated that rAc-PF may be an allergen in *Acanthamoeba*, but further studies needed to identify the mechanisms of allergenic reactions induced by Ac-PF.

## Author summary

Recently, the number of asthma patients have increased sharply. Among patients with asthma have a high serum IgE titer, but despite this, some of these patients do not react to known allergens in skin prick tests, that suggests the presence of unknown environmental allergens. The protozoa *Acanthamoeba* live in very diverse environment including water, soil, air and even human nasal cavities, throat, pharynx and lung. In previous study, *Acanthamoeba* could be a new aero-allergen. Patients who showed positive results for the skin-prick test response to *Acanthamoeba*, their serum could be cross-reacted with several pollen allergens as well as *Acanthamoeba* total proteins. Additionally, the patients have common antibodies reacted to the 13–15 kDa *Acanthamoeba* unknown allergen. Profilin, which is found in all eukaryotic organisms in most cells, is an actin-binding protein that interferes with nucleation and restructuring of new filaments. Recent studies showed that profilin functions as a pan-allergen recognized by IgE in approximately 20% of birch pollen and plant food allergic patients. In *Acanthamoeba*, two isoforms of profilin (Ac-PF) have been identified: profilin-I and profilin-II. We examined profilin from *Acanthamoeba* as a potential human airway allergic agent because of its molecular weight (13–14 kDa) and cross-reactivity with several pollen allergens in the skin prick test showing positive results for *Acanthamoeba* in chronic cough patients. In this study, we expressed recombinant Ac-PF (rAc-PF) protein using an *Escherichia coli* expression system and evaluated whether Ac-PF is an airway allergic agent using an asthma animal model. Our study showed that rAc-PF may be an allergen in *Acanthamoeba*, but further studies needed to identify the mechanisms of allergenic reactions induced by Ac-PF.

## Introduction

Allergic airway inflammation is characterized by the production of allergen-specific immunoglobulin (Ig) E and Th2 cytokines including interleukin (IL)-4, IL-5, and IL-13 which lead to the immune cell recruitment and sensitization of effector cells such as eosinophils, basophils, and mast cells [1]. These allergic reactions are typically induced by allergens. Marsh *et al*. described highly purified and well-characterized allergens such as the pollen of grasses, weeds, and trees, as well as house dust mites, fungal spores, and dander from animals [2].

*Acanthamoeba*, pathogenic and opportunistic free-living amoebae [3], is protozoa genus that can survive in various environments and is isolated from the soil, dust, air, water seawater, swimming pools, domestic tap water, and contact lenses and cases [4]. Additionally, excretory-secretory (ES) proteins from *Acanthamoeba* species contain strong proteases [5, 6]. Our previous studies demonstrated that *Acanthamoeba* may be aero-allergens [7, 8]. Six *Acanthamoeba* trophozoite intranasal (I.N.) treatments induced allergic airway inflammation in mice [7]. Moreover, patients showing positive results to the skin-prick test response to *Acanthamoeba* exhibited higher *Acanthamoeba*-specific IgE levels compared to other patients and healthy persons [8]. Interestingly, patients who showed a positive skin-prick test response to *Acanthamoeba* exhibited cross-reactivity with several pollen allergens, including willow tree, poplar, elm, oak, velvet grass, and cockroach. In western blot analysis, chronic cough patients IgE antibodies reacted with ~15-kDa components of *Acanthamoeba* [8]. We examined profilin from *Acanthamoeba* as a potential human airway allergic agent because of its molecular weight (13–14 kDa) and cross-reactivity with several pollen allergens in the skin prick test showing positive results for *Acanthamoeba* in chronic cough patients [8]. In *Acanthamoeba*, two isoforms of profilin (Ac-PF) have been identified: profilin-I and profilin-II [9, 10].

Profilin, which is found in all eukaryotic organisms in most cells, is an actin-binding protein that interferes with nucleation and restructuring of new filaments [11–13]. Recent studies showed that profilin functions as a pan-allergen recognized by IgE in approximately 20% of birch pollen and plant food allergic patients [14]. *BetvI*, which is one of the main causes of Type I allergic reactions, is an allergenic protein from the pollen of the white birch (*Betula verrucosa*) and related to IgE binding in more than 95% of birch pollen allergic patients [15, 16]. Valenta *et al*. prepared cDNAs encoding IgE-binding birch pollen protein that differed from *BetvI*. This protein appeared to act as an allergen in individuals allergic to pollens of grasses and weeds. Furthermore, IgE antibodies from birch allergic individuals showed cross-reactivity with human profilin [17]. Another study confirmed that 23% of 30 celery allergic patients were sensitive to profilin [18, 19].

In this study, we expressed recombinant Ac-PF (rAc-PF) protein using an *Escherichia coli* expression system and evaluated whether Ac-PF is an airway allergic agent using an asthma animal model.

## Methods

### *Acanthamoeba* cultivation and total protein extraction

*Acanthamoeba lugdunensis* KA/E2 strain, isolated from human cornea inflammation patient in Korea, it was maintained in PYG medium. The KA/E2 strain has the same molecular characteristics as the *A*. *lugdunensis* L3A strain (ATCC 50240) [20]. To obtain total protein, live trophozoites were incubated in PYG medium for one week at 25°C. Following centrifugation at 12,000g for 30 min, the total protein was extracted from the pellet according to protocol of manufacture (Cell lysis, ThermoFisher Scientific Co. Waltham, MA USA). After obtaining total proteins, the ToxinSensor Gel Clot Endotoxin Assay Kit (Gen-Script, Piscataway, New Jersey, USA) was used to eliminate endotoxins.

### Cloning, expression, and extraction of Ac-RF

To amplify full-length Ac-RF, we designed primers based on the *A*. *castellanii* profilin I gene (GenBank No. XP_004351646.1). The primer sequences were as follows: Forward; 5′-GGA ATT CCA TAT GTC CTG GCA GAC GTA CG-3′ Reverse; 5′-CCG CTC GAG AAA GCC CTG ACC GAT GA-3′. Total RNA was extracted from *Acanthamoeba sp*. KA/E2 trophozoite using 1 mL of LPS Solution (Biozol, Eching, Germany), and cDNA was synthesized using MMLV reverse transcriptase (Promega, Madison, WI, USA) according to the manufacturer’s protocols. After confirming the PCR product, the fragment was subcloned into the C-terminal His-tagged fusion protein vector pET-26b. The constructs were transformed into the expression host *E*. *coli* BL21 (DE3). Expression of rAc-PF was induced with 0.5 mM isopropyl-thio-β-D-galactopyranoside for 4 h. The *E*. *coli* cell pellets were resuspended in lysis buffer [50 mM Tris-HCl (pH 7.5), 200 mM NaCl, and 1 mM dithiothreitol]. After sonication cell suspensions on ice (Branson Sonifier 450, Branson Ultrasonics, Danbury, CT, USA), the resulting cell lysates were centrifuged at 10,000 ×*g* for 45 min to remove insoluble cellular debris. The soluble and insoluble portions were fractionated on 15% SDS-polyacrylamide gel electrophoresis (SDS-PAGE) gels and visualized by Coomassie blue staining. The supernatants were collected and used for protein purification. The His-tagged profilin fusion protein was applied to a Ni-NTA (Amersham Pharmacia Biotech, Amersham, UK) column for purification.

### Protease activity

To evaluate the protease activity of rAc-PF, zymogram analysis was performed according to a previous study [7]. Briefly, rAc-PF and KA/E2 crude extract samples were mixed with 5× zymogram loading buffer and loaded on gelatin-gel, and electrophoresis was performed at 125 V for 60 min at 4°C. After electrophoresis, the gelatin-gel was incubated 30 min in zymogram renaturation buffer. After development, the gel was stained with Coomassie Brilliant Blue R-250 overnight and then distained.

### Production of polyclonal antisera for rAc-PF

Four-week-old female Wistar rat was purchased from Samtako Co. (Gyeonggi-do, Korea). Rats were immunized subcutaneously with a combination of 500 μg (in 0.5 mL PBS) of rAc-PF and 0.5 mL Freund’s complete adjuvant (Sigma-Aldrich, St. Louis, MO, USA). After 2 weeks, a 2nd subcutaneous injection was performed with the same dose of rAc-PF and 0.5 mL Freund’s incomplete adjuvant. Two weeks after the final booster, rats were sacrificed and the serum was obtained.

### Western blotting

The rAc-PF and KA/E2 total proteins were loaded each well of a 12% acrylamide SDS-PAGE gel. The loaded proteins were transferred onto nitrocellulose membranes (Amersham Biosciences, Amersham, UK), and then blocked with 5% skim milk in TBS-T at 4°C overnight. After one day, the membrane was incubated with primary antibody in 5% skim milk in TBS-T at 4°C overnight. The secondary antibody, anti-rat IgG-horseradish peroxidase conjugate (Sigma) was reacted for 1 h at 24°C. Reactants were analyzed using ECL kit (Amersham Biosciences) using the LAS-3000 program.

### Experimental design for inducing allergic airway inflammation by rAc-PF

Six-week-old female C57BL/6 mice were purchased from Orient-bio Co. (Gyeonggi-do, Korea). The mice were maintained in a specific pathogen-free facility at the Institute for Laboratory Animals of Pusan National University during the experimental period. The mice were divided into 4 groups. Mice in the positive control group were intra-nasally administered 10 μg of *Aspergillus* protease (Sigma-Aldrich), a well-known substance that provoke allergic airway inflammation, 6 times at intervals of 2 days. Using the same protocol, mice in the other two groups were treated with each 10 and 50 μg of rAc-PF. On the day before sacrifice, airway hyperresponsiveness (AHR) was measured, and then the animals were sacrificed.

### AHR measurements

At 24 h after the last challenge, airway responsiveness was evaluated by measuring the change in lung resistance in response to aerosolized methacholine (Sigma-Aldrich) according to Kang et al. [21]. Briefly, to measure bronchoconstriction, the enhanced pause (PenH) was measured at baseline (PBS aerosol; control) and after exposure to increasing doses of aerosolized methacholine (0–50 mg/mL) using whole-body plethysmography (Allmedicus, Korea). In the plethysmography procedure, the mice were acclimated for 3 min, exposed to nebulized saline for 10 min, and treated with increasing concentrations (0, 12.5, 25, and 50 mg/mL) of methacholine using an ultrasonic nebulizer (Omron, Japan). After each nebulization, the PenH values measured every three minutes during the experimental period were averaged. Graphs were generated showing the PenH values in response to increasing methacholine concentrations for each dose-matched group of mice.

### Analysis of bronchoalveolar lavage fluid (BALF) and differential cell counting

To obtain the BALF, the tracheas of the anesthetized mice were exposed and cut just below the larynx. A flexible polyurethane tube (outer diameter; 0.4 mm, length; 4 cm; BD Biosciences, San Jose, CA, USA) attached to a blunt 24-gauge needle (Boin Medical Co., Seoul, Korea) was placed inside the trachea. Next, the lungs were lavaged once with 800 μL of sterile cooling phosphate-buffered saline (PBS). The BALF samples were centrifuged for 5 min at 1500 ×g 4°C. The supernatants were moved to new microcentrifuge tube and frozen at -70°C; the remaining cell pellets were resuspended in 100 μL of PBS. Total cells were counted using a hematocytometer. To observe the differential cell counts, the same number of BALF cells was centrifuged on the slides at 500 rpm for 5 min using a Cytospin apparatus (Micro 12TM, Hanil Co., Seoul, Korea). The slides were dried and stained with Diff-Quick solution (Sysmex Co., Kobe, Japan). At least 500 cells per slide were evaluated to analyze differential leukocyte counts.

### Lymphocyte and splenocyte preparation

Following sacrifice, the blood serum of mice was obtained by cardiac puncture and lung-draining lymph nodes (LLNs) and spleen were collected. The LLN in normal mouse (PBS treated mouse) is too small to get enough number of lymphocytes for ELISA experiment, therefore we used only LLN of rAc-PF and *Aspergillus* protease treated group for ELISA experiment. Each LLN and spleen were ground with MONOJECT and treated with ammonium chloride potassium (ACK) hypotonic lysis solution (Sigma-Aldrich) for 1 min at room temperature for red blood cells lysis. After lysis, lymphocytes and splenocytes were filtered through 100-μm meshes (Small Parts, Inc., Miramar, FL, USA) and then washed three times. Next, the cells were counted with a hemocytometer and plated in 48-well plate as 5 × 10^6^ cells/mL in RPMI 1640 containing 10% fetal bovine serum and penicillin/streptomycin. For CD3 stimulation analysis, 0.5 μg/mL anti-CD3 antibody was added to the plated cells. The plate was incubated at 37°C with 5% CO_2_. After 72 h incubation, the culture supernatants were collected and stored at –20°C for enzyme-linked immunosorbent assay (ELISA) [22].

### ELISA

ELISA was conducted to analyze the changes in IL-4, IL-5, and IL-13 levels in the BALF supernatant and stimulated culture supernatant of lymphocytes and splenocytes with an ELISA kit (ebioscience, San Diego, CA, USA). according to the manufacturer’s protocols. A 96-well immunoplate was coated with capture antibody and incubated overnight at 4°C. Plates were blocked with 1% bovine serum albumin for 1 h at 37°C. Test samples and standards were added to the wells and incubated overnight at 4°C. The plates were washed with 200 μL of PBS containing 0.1% Tween-20 5 times. Next, 100 μL of biotin-conjugated anti-mouse detection antibodies were added to each well and incubated for 1 h at room-temperature. The plates were washed as described above and avidin-conjugated horseradish peroxidase was added for 30 min incubation at room temperature in the dark. Tetra-methyl-benzidine (100 μL) was used to reveal the blue color and 100 μL of diluted H_2_SO_4_ was added as the stop solution (Merck, Darmstadt, Germany). The solution in each well changed from blue to yellow. The absorbance of the final reactant was measured at 450 nm using an ELISA plate reader.

### Lung histopathology and immunohistology scoring

Lung tissues were fixed in formalin solution for 24 h and embedded in paraffin wax. Thin sections of embedded lungs were stained with hematoxylin and eosin (H&E) to analyze inflammatory cell infiltration and periodic acid-Schiff (PAS) reagent was used to quantify goblet cells. The sections were visualized by microscopy. The prevalence and severity of peribronchial and perivascular inflammation were scored by as previously described [23, 24]. A grade of 0 was assigned when no inflammation was detectable, while grade 4 indicated a high percentage of airways and blood vessels in the section cuffing by inflammatory cells (0 = normal tissue; 1 = <25%; 2 = 25–50%; 3 = 51–75%; 4 = >75%). Severity scoring was based on the thickness of the bronchi or vessels surrounded inflammatory cells (0 = no cells; 1 = 1–3; 2 = 4–6; 3 = 7–9 cells thickness; 4 = 10 or more cells thick). To quantify goblet cell metaplasia, the percentage of PAS-positive cells in hyperplasia areas was examined from 8 tissue sections per mouse.

### *In vitro* stimulation to analyze Th2-related gene expression

Mouse lung epithelial cells (MLE12) were obtained from ATCC (Manassas, VA, USA); 3 × 10^5^ MLE12 cells were plated in 24-well plates and incubated overnight at 37°C. The cells were stimulated with rAc-PF and *Aspergillus* proteins for 3 h. Next, MLE12 cells were collected with 1 mL of QIAzol (Qiagen, Hilden, Germany), and RNA extraction was conducted in accordance with the manufacturer’s protocols for transcription of 2 μg of RNA. Real-time PCR was performed to determine the levels of macrophage-derived chemokine (MDC; CCL22), eotaxin (CCL11), thymic stromal lymphopoietin (TSLP), and IL-25 RNA. GAPDH was used as an internal reference. The primers and PCR conditions used have been described previously [25, 26].

### Statistical analysis

All experiments were conducted three times for statistical analysis. The mean ± SD was calculated from data collected from different mice. Significant differences were determined using *t*-tests (and nonparametric tests) of variance. Statistical analysis was performed with GraphPad Prism 5.0 software (GraphPad Software Inc., La Jolla, CA, USA).

### Ethics statement

The study was performed with approval from the Pusan National University Animal Care and Use Committee (IACUC protocol approval; PNU-2016-1358), in compliance with “The Act for the Care and Use of Laboratory Animals” of the Ministry of Food and Drug Safety, Korea. All animal procedures were conducted in a specific pathogen-free facility at the Institute for Laboratory Animals of Pusan National University.

## Results

### Expression of recombinant Ac-PF (rAc-PF) using *E*. *coli* expression system

**Fig 1:**
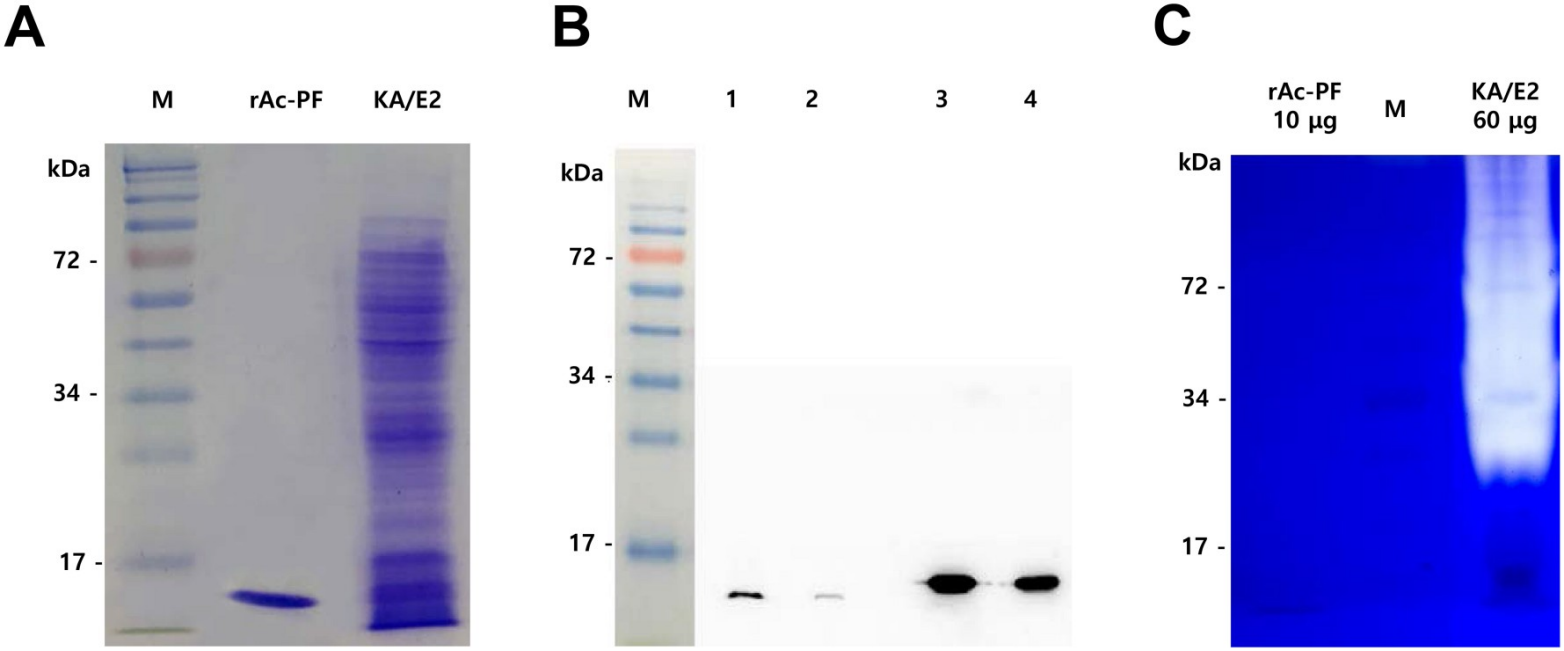
Expression of rAc-PF and evaluation of Ac-PF expression in *Acanthamoeba*. (A) First, 3 μg of rAc-PF and 120 μg of KA/E2 total proteins were loaded into each well for 12% acrylamide SDS-PAGE. (B) After separation, the proteins were transferred to a nitrocellulose membrane and anti-rAc-PF and Rat IgG-horseradish peroxidase were reacted as primary and secondary antibodies (each 1:1000 diluted). Lane M; protein molecular marker, lane 1; 100 μg of KA/E2 total proteins, lane 2; 20 μg of KA/E2 total proteins, lane 3; 5 μg rAc-PF, lane 4; 2 μg rAc-PF). (C) The samples were incubated for 2 h and assayed by zymography by 0.1% gelatin SDS-PAGE. A clear background against dark-blue where the protease digested the substrate indicated protease activity, (M; protein molecular marker, rAc-PF, recombinant Ac-PF, KA/E2, total protein of KA/E2).

After expression of rAc-PF, an approximately 15-kDa recombinant protein was detected by SDS-PAGE (Fig 1A). To confirm the expression of Ac-PF in *Acanthamoeba*, a polyclonal anti-rAc-PF antibody was produced and reacted with *Acanthamoeba* total proteins. An approximately 13-kDa protein in the *Acanthamoeba* total protein reacted with the anti-rAc-PF antibody. Because rAc-PF has 6 histidine tags, the size was slightly larger than Ac-PF alone (Fig 1B). To evaluate the protease activities of rAc-PF, we conducted zymogram analysis and compared the results with those of the total protein of *Acanthamoeba* KA/E2. The KA/E2 total protein showed strong protease activity, while rAc-PF did not show this activity (Fig 1C).

### rAc-PF provoked allergic airway inflammation

**Fig 2:**
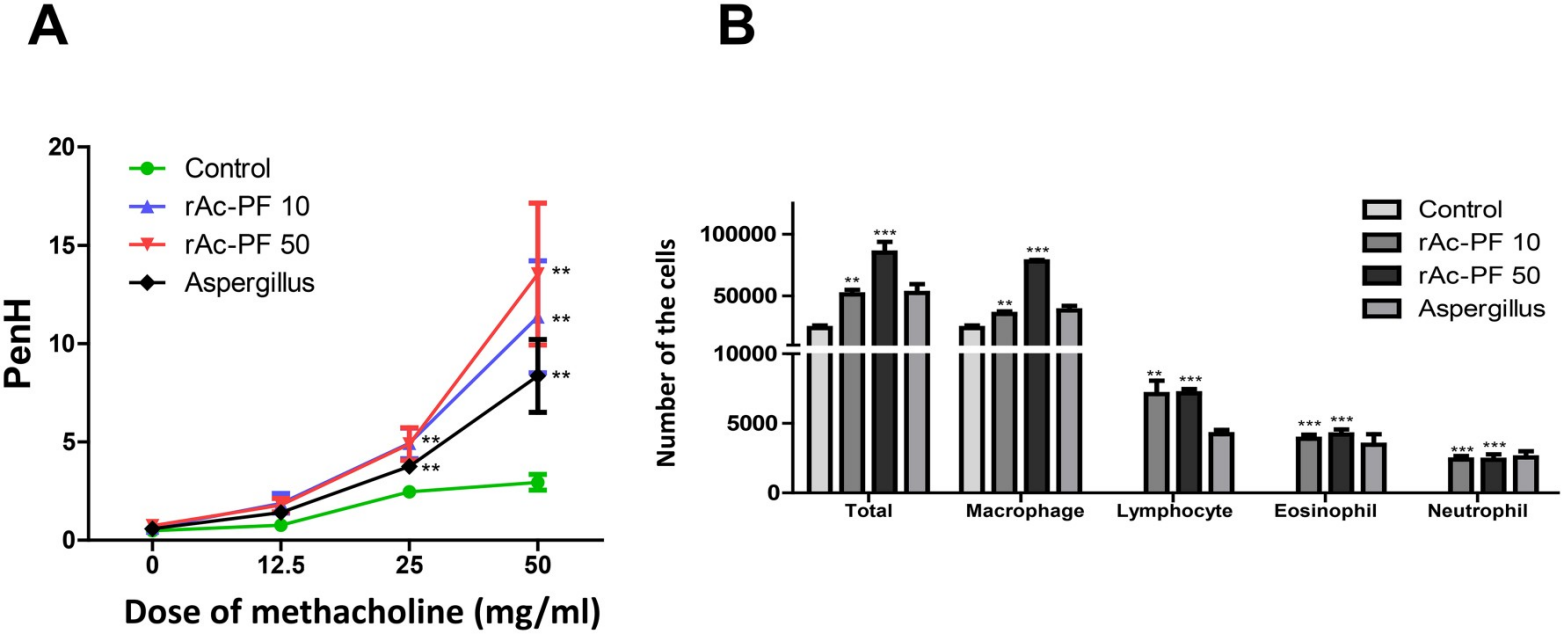
Airway hyperresponsiveness and differential cell counts in BALF. Allergic airway inflammation was induced by 6 intranasal (I.N.) administrations of 10 or 50 μg rAc-PF in mice. (A) Measurement of airway hyperresponsiveness to methacholine (0–50 mg/mL). (B) After the BALF was obtained by lavage through the tracheas with 800 μL of sterile PBS using a blunt 24-gauge needle, cells were isolated by centrifugation. Cells were stained with Diff-Quick solution (Sysmex Co., Shanghai, China) and then differential cells were counted. (Control; PBS I.N. treatment, rAc-PF 10; 10 μg rAc-PF I.N. treatment, rAc-PF 50; 50 μg rAc-PF I.N. treatment, Aspergillus; 10 μg *Aspergillus* protease I.N. treatment, *p < 0.05, **p < 0.01, ***p < 0.001, n = 6 mice/group, 3 independent experiments).

**Fig 3:**
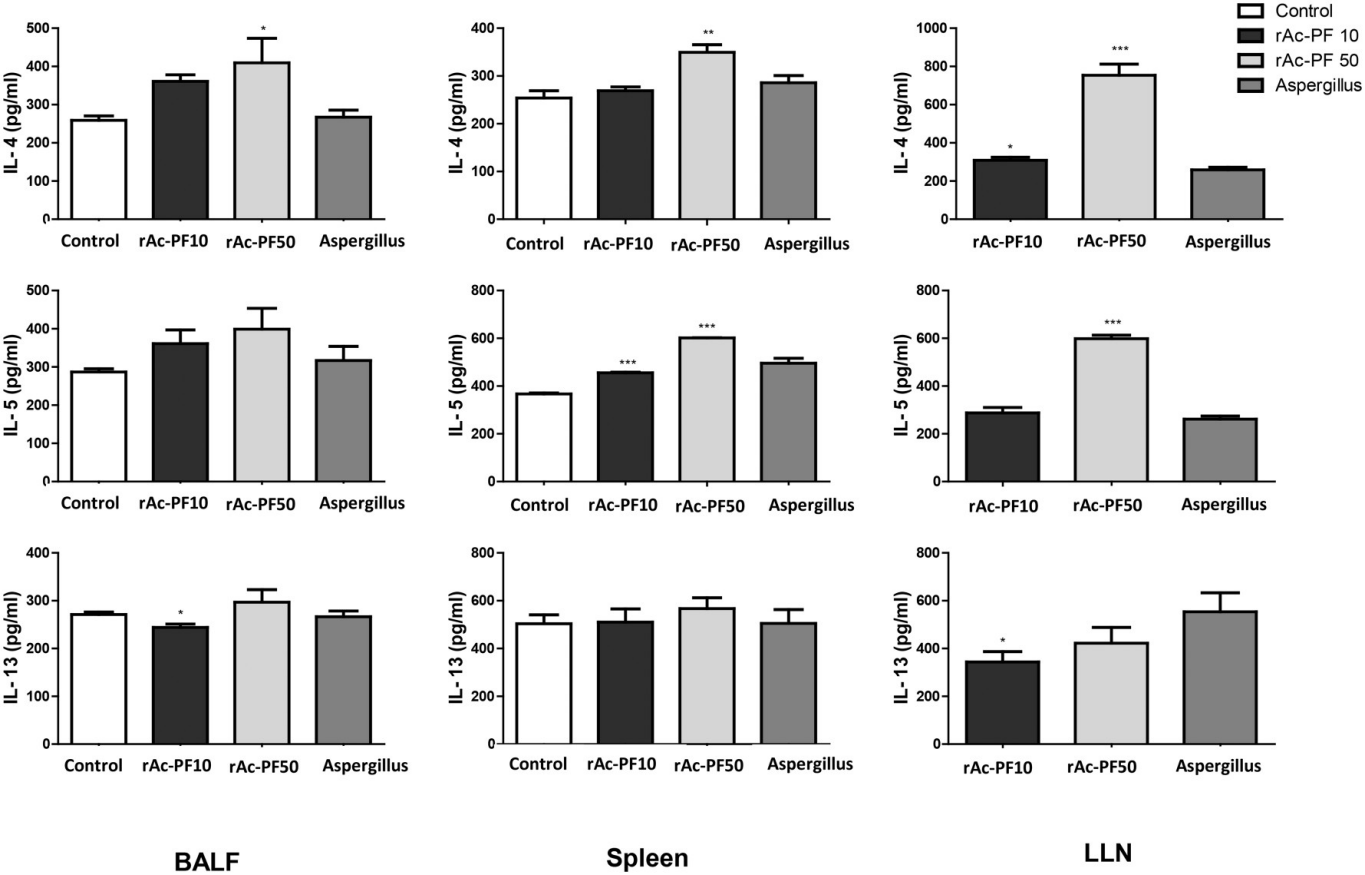
Expression levels of Th2 cytokines in various immune organs after treatment with rAc-PF. Th2 cytokine (IL-4, IL-5, and IL-13) concentrations in the BALF and culture supernatants of CD3-stimulated lymphocytes from LLN and splenocytes were measured by ELISA. The plates were read at 450 nm on a standard ELISA reader, VICTOR 3 (Each *p*-value was determined based on *t*-test methods compared to control. *p < 0.05, **p < 0.01, ***p < 0.001, n = 6 mice/group, 3 independent experiments, Control; PBS I.N. treatment, rAc-PF 10; 10 μg rAc-PF I.N. treatment, rAc-PF 50; 50 μg rAc-PF I.N. treatment, Aspergillus; 10 μg *Aspergillus* protease I.N. treatment).

To evaluate the effect of Ac-PF on allergic airway inflammation in mice, *Aspergillus* protease (Sigma-Aldrich), which is well known as a strong allergen, and rAc-PF were treated intranasally 6 times in each mouse. Airway hyperresponsiveness to a 0–50 mg/mL dose of methacholine was increased in the rAc-PF inoculated mouse group (Fig 2A). Additionally, the numbers of eosinophils, neutrophils, and lymphocytes were increased in the BALF of rAc-PF-treated mice (Fig 2B). The levels of Th2 cytokines, IL-4, IL-5, and IL-13, in the BALF and culture supernatants of the LLN and spleen were increased in the rAc-PF-treated groups compared to in the control group (Fig 3).

### Treatment of rAc-PF induced inflammatory cell infiltration and hyperplasia of goblet cells in the lungs

**Fig 4:**
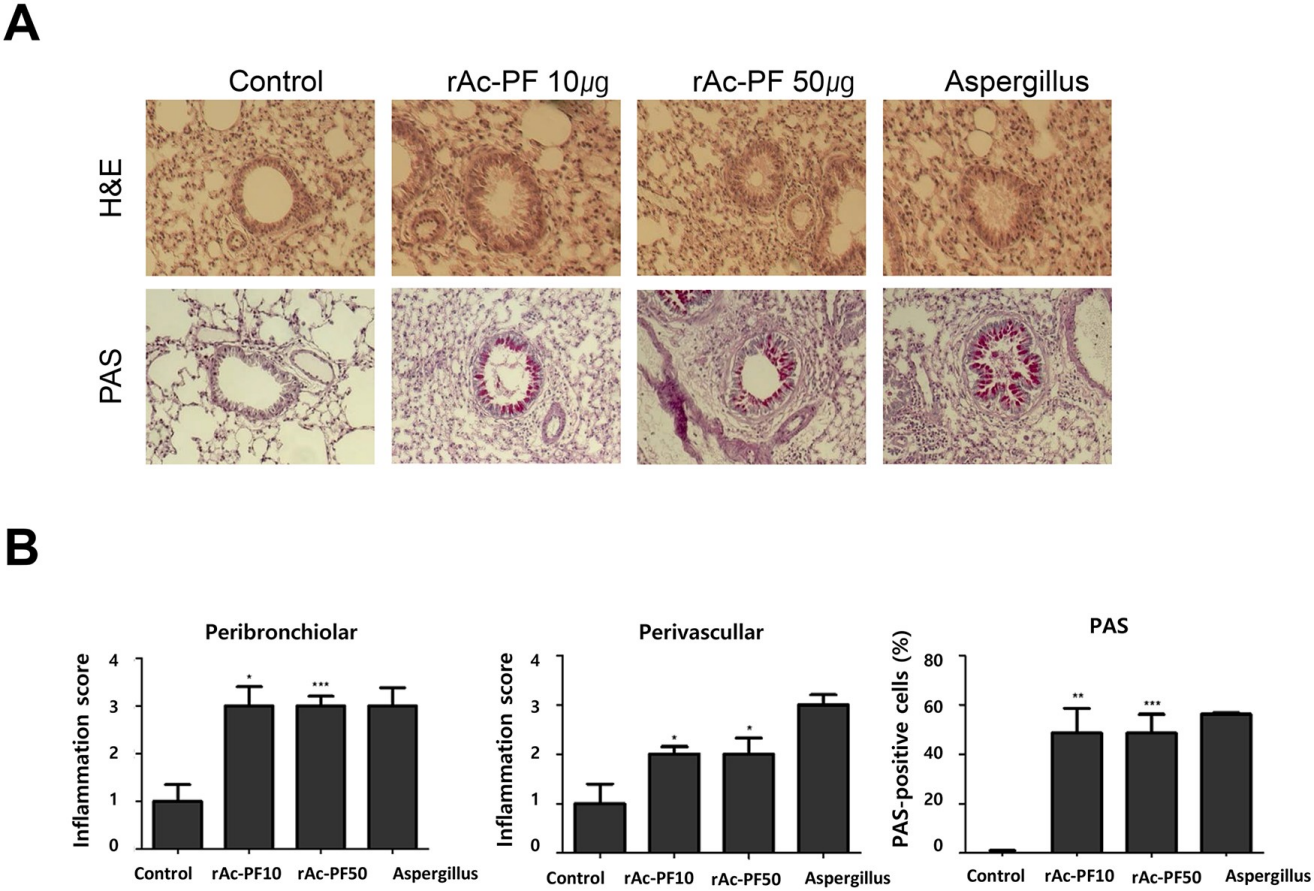
Representative histopathological changes after rAc-PF treatment. (A) Lung tissue inflammation observed on images of lung sections after H&E and PAS staining. (B) Representative of inflammation scoring and PAS-positive cells (mean ± SEM). A grade of 0, when no inflammation was detectable, to a grade 4 for high percentages of airways and blood vessels in section cuffing by inflammatory cells (0 = normal tissue; 1 = <25%; 2 = 25–50%; 3 = 51–75%; 4 = >75%). Severity scoring was based on the thickness of bronchi or vessels surrounded inflammatory cells (0 = no cells; 1 = 1–3; 2 = 4–6; 3 = 7–9 cells thick; 4 = 10 or more cells thick). Means of *p*-value was measured to compare to control (*p < 0.05, **p < 0.01, ***p < 0.001, n = 6 mice/group, 3 independent experiments, Control; PBS I.N. treatment, rAc-PF 10; 10 μg rAc-PF I.N. treatment, rAc-PF 50; 50 μg rAc-PF I.N. treatment, Aspergillus; 10 μg *Aspergillus* protease I.N. treatment).

To examine whether Ac-PF influences bronchial trees, we focused on histological changes in the lung tissue. Lungs from treated and non-treated mice were isolated and stained with H&E to analyze inflammatory cell infiltration and PAS to quantify goblet cells. The lungs of rAc-PF and *Aspergillus* protease-treated group mice showed dramatic immune cell infiltration surrounding the bronchial trees and vessels, enhanced mucin production, and metaplasia of lung epithelial cells and goblet cells (Fig 4A). For histopathological analysis, the inflammation score was determined as the prevalence and severity of inflammation. Peri-bronchiolar and peri-vascular inflammation scores were significantly higher compared to in non-treated mice. Additionally, PAS-positive cells (goblet cells) were significantly increased compared to in the control group (Fig 4B).

### Treatment of rAc-PF induced Th2 chemokines gene expression in mouse lung

**Fig 5:**
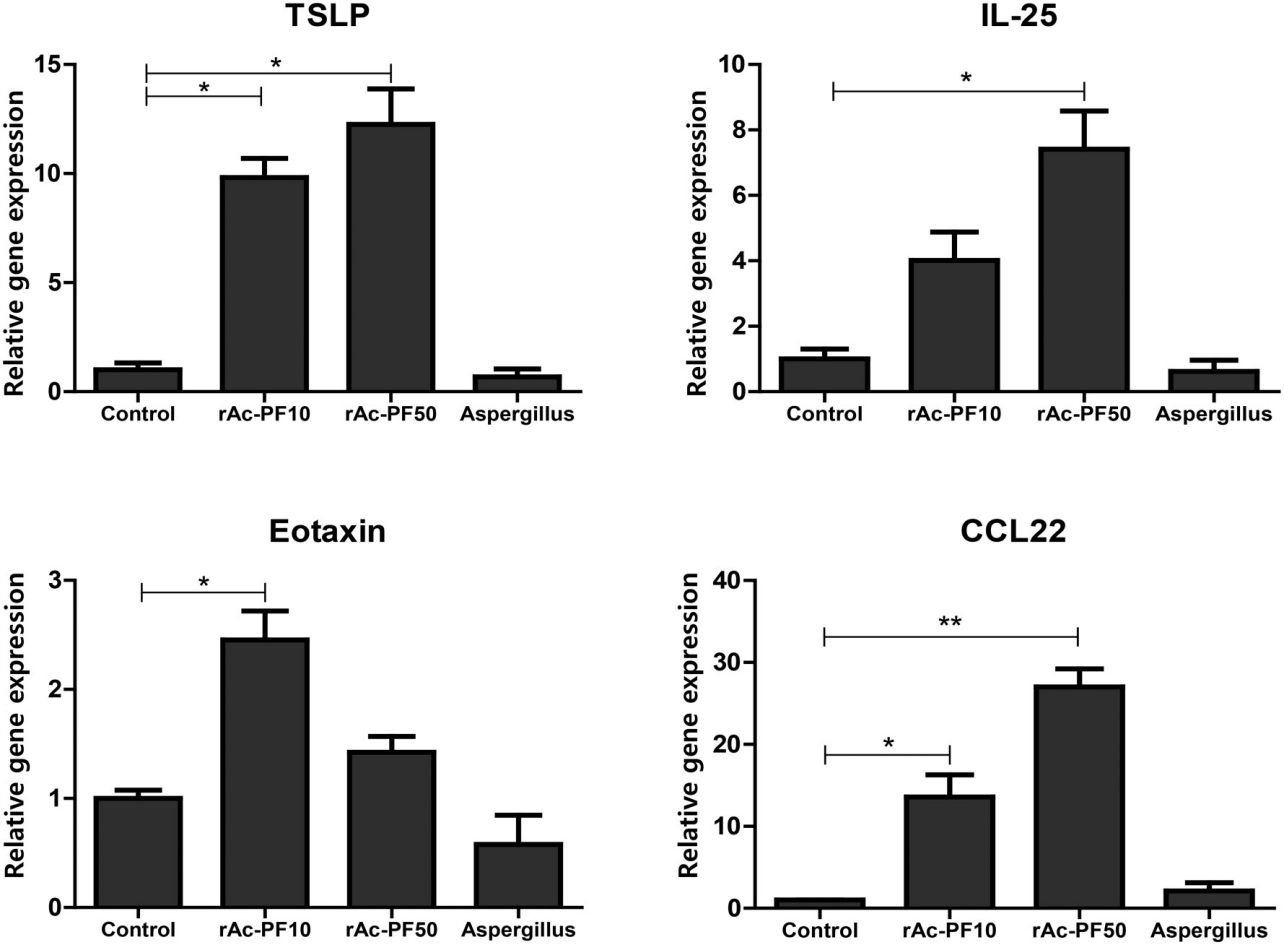
Treatment with rAc-PF elicited Th2-related chemokine gene expression. Th2-related chemokine gene expression levels of TSLP, MDC, eotaxin, and IL-25 were measured in MLE12 cells after incubation with rAc-PF protein and *Aspergillus* proteins for 3 h. Means of *p*-value were measured for comparison to control (Control; PBS treatment, rAc-PF 10; 10 μg rAc-PF treatment, rAc-PF 50; 50 μg rAc-PF treatment, Aspergillus; 10 μg *Aspergillus* protease treatment, *p < 0.05, **p < 0.01, ***p < 0.001, 3 independent experiments).

To evaluate whether rAc-PF influences lung epithelial cells, Th2 chemokine gene expression levels were examined after treating MLE12 cells with rAc-PF. After 3hr later, the treatment significantly increased the levels of MDC, TSLP, eotaxin, and IL-25 gene expression (Fig 5). However, expression levels of those genes of *Apergillus* protease treated cell were not increased at that time point. These chemokines are known as essential for the initiation and expansion of the Th2 response in lung epithelial cells.

## Discussion

In this study, we investigated the ability of Ac-PF to provoke allergic airway inflammation in an animal model. Ac-PF-treated mice showed similar symptoms to asthma, including goblet cell and immune cell infiltration, increased mucin production, and hyperplasia of respiratory epithelial cells, which causes the airway tract to become narrow, leading to increased airway hyperresponsiveness in a methacholine dose-dependent manner.

*Acanthamoeba* are free-living, amphizoic and opportunistic protozoa that are common in nature [27]. A previous study demonstrated that *Acanthamoeba* trophozoites induced allergic airway inflammation in mice by inducing a Th2 response [28]. These allergic airway inflammation effects were closely related to the protease activity of excretory-secretory proteins (ESP) of *Acanthamoeba* [7]. Several studies showed that protease activity was related to allergens and led to morphologic changes and cytokine production [29]. Kheradmand et al. found that inactivated protease allergen fragments showed no allergenic potency, demonstrating that active protease is essential for the allergen effects [30]. However, we found that protease activity was not the only essential factor in the allergic airway inflammation effect of *Acanthamoeba*. Although most effects were significantly reduced after blocking the protease activity of ESP, some allergic symptoms and effects were observed after treatment with boiled ESP [9]. We predicted that protease activity acts as one of the strongest allergic factors, and other allergens may be present in *Acanthamoeba* ESP or their extracts. rAc-PF may be one of the protease activity free allergens, as some agents did not exhibit protease activity, but still exhibited allergic inflammation ability (Fig 1C).

Profilin is intermediate or major allergen in pollen and foods [31, 32]. Carlsson et al. first identified profilin as a profilamentous complex of actin-associated protein essential for the spatial regulation of actin microfilament growth. This is an essential process in cellular movement and cell shape changes [33–35]. It was also proposed that profilin elicits Type I allergic reactions because of the three-dimensional structures of some allergenic profilins [36, 37]. Fedorov et al. reported the allergen cross-reactivity, crystal structure, and IgE-epitope mapping of birch pollen profilin. The epitopes reside in conserved sequences, thus providing an explanation for the cross-reactivity [38]. Park et al. detected IgE antibodies in patients with a positive skin-prick test to pollen who also reacted with *Acanthamoeba* ES proteins and showed positive results in patients positive to *Acanthamoeba* [8]. Although profilin is regarded as an allergen, its role in allergic symptoms is controversial. Many studies have described sensitization to profilin and cross-reactivity of IgE with this allergen [16, 17, 31, 38–40], but the sensitization and immunological cross-reactivity do not always lead to allergy.

Allergic airway inflammation is induced by allergens through several mechanisms. For example, an allergenic substrate can activate dendritic cells and induce IL-25, TSLP, and allergy-related chemokines [41]. In this study, we found that Th2 chemokine gene expression in mouse lung epithelial cells was significantly increased on 3hr after rAc-PF treatment (Fig 5), and Th2 cytokine expression levels in the BALF, MLN, and spleen were increased by repeated nasal treatment with rAc-PF in mice (Fig 3). In previous studies, *Aspergillus* can elicit Th2 chemokine genes *in vivo* experiments, we also found airway inflammation by *Aspergillus* treatment (Figs 3 and 4). Although the level of chemokine genes related with Th2 in *Aspergillus* proteinase treated mouse lung epithelial cell was not elevated on 3 hrs after treatment in this study, they could elevate the gene expression on more early time [25]. Previous studies revealed that *Acanthamoeba* ESP activated dendritic cells, increasing the differentiation of naïve CD4^+^ T cells into T helper type 2 (Th2) cells [28].

In conclusion, rAc-PF increased the numbers of lymphocytes, eosinophils, and neutrophils in the BALF and levels of Th2 cytokines. Further studies are needed to determine the mechanisms used by Ac-PF to enhance allergenic reactions. Additionally, alterations in immunocytes *in vitro* and how Ac-PF influences activation of innate immunity should be examined, which may facilitate diagnosis and the development of new treatments for allergic diseases in the future.
